# The epidemiology of notifiable diseases in Australia and the impact of the COVID-19 pandemic, 2012–2022

**DOI:** 10.1186/s44263-023-00029-y

**Published:** 2024-01-02

**Authors:** Asma Sohail, Allen C. Cheng, Sarah L. McGuinness, Karin Leder

**Affiliations:** 1 School of Public Health and Preventive Medicine, Monash University, 553 St Kilda Road, Melbourne, VIC 3004 Australia; 2 Department of Infectious Disease, Grampians Health Service, 1 Drummond Street North, Ballarat, VIC 3350 Australia; 3 Department of Infectious Diseases, Alfred Hospital, 55 Commercial Road, Melbourne, VIC 3004 Australia; 4 Monash Infectious Diseases, Monash Health, 246 Clayton Road, Clayton, VIC 3168 Australia; 5 Victorian Infectious Disease Service, Royal Melbourne Hospital, 300 Grattan Street Parkville, Victoria, 3050 Australia

**Keywords:** Public health, Epidemiology, Notifiable diseases, Infectious disease, Surveillance, COVID-19

## Abstract

**Background:**

Infectious disease surveillance tracks disease epidemiology and informs prevention and control. Public health measures implemented in Australia during the COVID-19 pandemic (2020 to 2022) affected infectious disease epidemiology. We examined notifiable disease epidemiology in Australia from 2012 to 2022, evaluating disease trends and pandemic impacts.

**Methods:**

We analysed case notifications supplied to the Australian National Notifiable Disease Surveillance System (NNDSS) from 1 January 2012 to 31 December 2022. The annual incidence and notification incidence trends were calculated and the average changes in annual incidence were investigated by Poisson regression.

**Results:**

Over the study period, there were 14,087,045 notifications of 68 diseases. Respiratory diseases were the most commonly notified disease group (83% of all notifications) and vector-borne diseases the least (< 1%). The ten highest-incidence diseases comprised 97% of all notifications over the study period, with COVID-19 alone accounting for 72%. Notifications were most common among the 20–39-year age group (37%). From 2012–2019, notification incidence of gastrointestinal, respiratory and sexually transmissible infections increased, whereas for bloodborne viral hepatitis, vector-borne diseases and imported diseases it decreased. From 2020–2021, average notification incidence of most non-COVID-19 respiratory diseases decreased compared to the 2012–2019 period; sexually transmissible infections notification incidence remained fairly stable; notification incidence of some gastrointestinal diseases increased while others decreased; and notification of imported diseases markedly decreased. A rebound in notification incidence was seen for most diseases in 2022.

**Conclusions:**

Prior to the COVID-19 pandemic, most notifiable diseases had increasing notification incidence, except for bloodborne viral hepatitis, vector-borne diseases and imported diseases. COVID-19-related public health measures had variable impacts on notifiable diseases.

**Supplementary Information:**

The online version contains supplementary material available at 10.1186/s44263-023-00029-y.

## Background

Infectious disease surveillance systems enable detailed analysis and interpretation of epidemiological data to inform public health policy and minimise morbidity and mortality [1]. In Australia, notification of selected infectious diseases is required by public health legislation across all states and territories [2]. Each jurisdiction defines its own notification list and receives data from doctors and/or laboratories. Jurisdictions then forward de-identified notification data for diseases on the National Notifiable Disease List (NNDL) (Additional file 1: Table S1) to the National Notifiable Disease Surveillance System (NNDSS), a passive surveillance system operational since 1991.

Since early 2020, the coronavirus disease-2019 (COVID-19) pandemic has caused social and economic disruption in Australia and globally [3, 4]. The non-pharmaceutical public health measures subsequently introduced to prevent and control COVID-19 (such as border closures, jurisdictional lockdowns, and mandatory mask-wearing) have affected infectious disease transmission dynamics, along with healthcare-seeking behaviour, healthcare access, and testing strategies, all of which may impact disease notification rates [5–7].

Studies from other countries have highlighted changes in infectious disease notification trends in the setting of the COVID-19 pandemic. In China, respiratory and gastrointestinal disease notifications declined significantly in 2020 and non-respiratory disease notifications rebounded when public health control measures were relaxed at the end of 2020 [8]. Countries in Europe reported similar decreases in respiratory, gastrointestinal, and vector-borne disease notifications in 2020 [9, 10], with notifications rebounding when public health control measures were lifted. In Taiwan, while notifications for most respiratory and imported infectious diseases declined in 2020, the overall incidence of sexually transmissible infections increased [11].

Limited data from Australia from early in the pandemic and for limited jurisdictions [6, 12] also suggest that the COVID-19 pandemic and related public health control measures impacted notifiable disease trends. However, an assessment of the differential impacts of COVID-19-related control measures on a range of disease notifications is currently lacking.

A previous analysis of the first 21 years of NNDSS data (1991–2011) [1] provided a comprehensive overview of the historical epidemiology of notifiable diseases in Australia, describing disease trends over time. Here, we aim to conduct a detailed analysis of NNDSS data from 2012 to 2022 to examine notifiable disease trends over the last decade, including the impacts of the COVID-19 pandemic.

## Methods

We analysed case notifications of nationally notified diseases to the NNDSS from 1 January 2012 to 31 December 2022 according to their diagnosis date. We excluded human immunodeficiency virus (HIV) and Creutzfeldt-Jacob disease (CJD) as they are monitored under different national surveillance systems. Case definitions for all notifiable diseases are developed by the Communicable Disease Network of Australia (CDNA) and have been in use since 2004, and undergo subsequent periodic revisions and updates [13]. The year that a disease became notifiable is listed as 1991 for those that were nationally notifiable when NNDSS began in 1991 [14] (Additional file 1: Table S1); however, diseases introduced after 1991 might have cases notified to NNDSS prior to becoming nationally notifiable. Some diseases became nationally notifiable during the study period and some diseases are not notifiable in all jurisdictions or have become notifiable in different jurisdictions at different times. We categorised each notifiable disease into one of six groups based on the main mode of transmission/acquisition: gastrointestinal, respiratory, vector-borne diseases, bloodborne viral hepatitis, sexually transmissible infections, and others. We also created an additional group of primarily imported diseases to enable analysis of travel-related infections; there is an overlap of individual diseases in this group with other disease groups (Additional file 1: Table S1). We divided the study period into two sub-periods (2012–2019, 2020–2022) to enable analysis of the impact of the COVID-19 pandemic and associated public health prevention measures on notifications.

We report the number and annual incidence of notified cases nationally and by jurisdiction. For all-cause, disease-group, and disease-specific incidence calculations, all notified cases were included and Australian Bureau of Statistics (ABS) resident population estimates on 30 June for each study year were used [15]. For individual diseases, incidence calculations were confined to years the disease was notifiable, either nationally or in a jurisdiction. Misclassified notification data were excluded from analyses. Formal ABS national, jurisdictional, and age-based population estimates were not available for June 30, 2022; national and jurisdictional populations were instead estimated based on available 2022 data till 31 March and projected data from the ABS population clock for 30 September 2022 [15]. Age was divided into five groups: < 5 years, 5–19 years, 20–39 years, 40–59 years, and ≥ 60 years. Age-specific population estimates from 31 March 2022 were used for age-based incidence calculations, due to there being no age-based data available for 30 September 2022 [15].

Average changes in annual notification incidence over the study period were investigated by Poisson regression for diseases with ≥ 400 notifications. This was done for disease groups and individual diseases, as well as by jurisdictions and age groups. Results were considered statistically significant if *p* < 0.05.

The notification incidence trend was calculated for six disease groups, excluding respiratory diseases. The number of notifications averted during the pandemic was estimated by assuming the counterfactual was represented by an extension of the pre-pandemic trend and subtracting the observed from the expected number of notifications. Influenza notifications contributed substantially to respiratory disease notifications prior to 2020; due to significant year-to-year variation in influenza notifications, a pre-pandemic trend could not be established with confidence. Therefore, an estimation of the number of respiratory notifications averted due to the COVID-19 pandemic was not made.

NNDSS data were provided by the Australian Government’s Office of Health Protection on behalf of Communicable Diseases Network Australia (CDNA) jurisdictional members in July 2023.

The project was approved by the Monash University Human Research Ethics Committee (MUHREC; project #28,955) and CDNA jurisdictional members. Data were analysed using STATA version 15 (College Station, TX: StataCorp LLC).

## Results

The NNDSS recorded 14,087,045 notifications of 68 diseases from 1 January 2012 to 31 December 2022 (Table 1). Respiratory diseases were the most commonly notified disease group, comprising 83% of all notifications over the study period; vector-borne diseases were the least common (< 1%; Table 1). Notification numbers without inclusion of COVID-19 are presented in Additional file 1: Table S2. COVID-19, notifiable from 2020, was the most commonly notified disease overall (10,124,662 notifications [72.0%]) and comprised the majority of notifications in 2022 (9,557,691 [94%]; Table 2). The ten highest-incidence diseases over the study period were COVID-19, influenza, chlamydia, respiratory syncytial virus (RSV), campylobacteriosis, gonorrhoea, varicella zoster virus (VZV; shingles and unspecified combined), salmonellosis, pertussis, and hepatitis C, collectively comprising 97% of all notifications (Table 2). Fewer than twenty notifications were received for seven diseases, and no notifications were received for eleven diseases (Table 2).

**Table 1:** Number, demographics, and crude incidence of case notifications by disease group and jurisdiction, Australia 2012–2022

	**Notifications**		**Sex**		**Age group (years)**					**Crude incidence (100,000 per year) 2012–2022**	
	***N***	**(%)**	**Male (%)**	**Female (%)**	** < 5 (%)**	**5–19 (%)**	**20–39 (%)**	**40–59 (%)**	** ≥ 60 (%)**	**Mean**	**Range**
**All notifications**	14,087,045	100	45	50	5	19	37	24	15	5013	994–39,089
**Disease group**											
Gastrointestinal	566,595	4	52	48	18.4	15.1	26.5	19.2	20.8	209	154–254
Respiratory	11,641,375	82.6	44	50	5.6	20.1	33.1	25.2	15.9	4108	202–38,141
Sexually transmissible	1,358,281	9.6	53	47	0.1	16.1	70.9	11.4	1.5	503	441–594
Bloodborne viral hepatitis	171,842	1.2	62	38	0.2	2.5	47.4	38.1	11.8	64	47–76
Vector-borne	80,654	0.6	48	52	0.3	6.1	32.9	40.5	20.2	30	14–51
Other	268,298	1.9	46	54	1.1	9.1	23.8	28	38	99	63–125
Imported	23,622	0.2	55	45	3.3	13.4	42.7	30.1	10.5	9	0–13
**Jurisdiction**											
Australian Capital Territory	286,695	2	47	50	5.4	18.6	39.3	24	12.7	5947	912–51,953
New South Wales	4,794,516	341	49	51	5.4	19.7	35.8	24.1	15	5359	685–43,234
Northern Territory	198,091	1.4	47	53	7.4	20.7	42.3	20.9	8.7	7279	2735–45,904
Queensland	1,986,999	14.1	45	52	5.6	16.9	37.5	23.1	16.4	3521	1222–21,173
South Australia	1,194,180	8.5	47	52	6.1	19.6	33.1	23.5	17.4	6098	1071–49,643
Tasmania	202,936	1.4	45	54	5.4	16.1	33.7	23.2	21.6	3567	803–26,296
Victoria	3,743,878	26.6	47	52	5.1	18.3	37.6	23.9	14.8	5222	955–41,678
Western Australia	1,679,750	11.9	30	33	5.3	20.6	35.9	24.7	13	5642	1053–48,128

*NB* overlap of individual diseases within the ‘imported’ disease group with other disease groups

Age group missing data: 0.1% for all notifications with COVID-19 notifications accounting for 92% of missing data; 0.5% for Western Australia, 0.5% for Queensland; 0.3% for South Australia, Victoria

Sex missing data: 5% for all notifications, 6% for respiratory diseases, 3% for the Australian Capital Territory. Queensland; 1% South Australia, Tasmania, Victoria; 37% for Western Australia with COVID-19 notifications accounting for > 99% of the missing data

**Table 2:** Notification incidence (per 100,000 population per year) by year for disease groups and individual diseases, Australia 2012–2022

	**Notifications**	**Annual incidence**											**Mean incidence over different time periods**			**Change in annual incidence (2012–2019)**	
		**2012**	**2013**	**2014**	**2015**	**2016**	**2017**	**2018**	**2019**	**2020**	**2021**	**2022**	**Mean incidence (2012–2019)**	**Mean incidence (2020–2021)**	**Mean incidence (2012–2022)**	**Ave annual change in incidence (%)**	**95% CI; *p* value**
**All diseases (includes COVID-19)**	14,087,045	1100	994	1188	1420	1424	2110	1324	2363	1132	3002	39,089	1490	2067	5013		
**All diseases (excludes COVID-19)**	3,962,383	1100	994	1188	1420	1424	2110	1324	2363	1018	909	2234	1490	964	1462	11.36	5.69–17.33; < 0.001
**Respiratory diseases (includes COVID-19)**	11,641,375	331	202	365	545	491	1105	325	1321	236	2126	38,141	586	1181	4108		
**Respiratory diseases (excludes COVID-19)**	1,516,713	331	202	365	545	491	1105	325	1321	122	33	1286	586	78	557	22.98	9.95–37.54; < 0.001
COVID-19	10,124,662	N/A	N/A	N/A	N/A	N/A	N/A	N/A	N/A	114	2093	36,855		1104	13,021		
*H. influenzae* type b	196	0.1	0.1	0.1	0.1	0.1	0.1	0.1	0.1	0.1	0.1	0.05	0.1	0.1	0.1		
Influenza	1,211,009	197	122	288	422	376	1,022	236	1237	83	3	900	487	43	444	29.94	− 4.97–1.83; 0.35
Legionellosis	5094	1.7	2.2	1.8	1.5	1.5	1.6	1.8	1.7	2.1	2.3	2.6	1.7	2.2	1.9	− 1.63	− 3.08 – − 0.16; 0.03
Ornithosis	398	0.3	0.2	0.2	0.1	0.1	0.1	0.04	0.1	0.2	0.1	0.2	0.1	0.2	0.1		
Pertussis	132,373	106	54	51	95	83	50	50	47	13	2	2	67	8	50	− 7.63	− 13.70 – − 1.12; 0.02
Invasive pneumococcal disease	18,620	8	7	7	6	7	8	8	8	4	5	7	7	5	7	2.55	− 0.73–5.94; 0.13
Respiratory Syncytial Virus	95,413	N/A	N/A	N/A	N/A	N/A	N/A	N/A	N/A	N/A	6	362			184		
Diphtheria	89	0	0.01	0.01	0.01	0.03	0.03	0.04	0.03	0.04	0.03	0.1	0.02	0.04	0.03		
Tuberculosis	15,293	6	5	6	5	6	6	6	6	6	6	5	6	6	6	0.77	− 0.28–1.83; 0.15
Invasive meningococcal disease	2127	1	0.6	0.7	0.8	1	1.5	1.1	0.8	0.4	0.3	0.5	1	0.3	0.8	5.12	− 3.12–14.06; 0.23
Measles	1369	0.9	0.7	1.4	0.3	0.4	0.3	0.4	1.1	0.1	0	0.03	0.7	0.1	0.5	− 4.53	− 19.71–13.51; 0.60
Mumps	3890	0.9	0.9	0.8	2.7	3.3	3.3	2.5	0.7	0.6	0.1	0.2	1.9	0.3	1.5	9.10	− 8.92–30.68; 0.34
Rubella	158	0.2	0.1	0.1	0.1	0.1	0.04	0.04	0.1	0.01	0.01	0.01	0.1	0.01	0.06		
Varicella zoster virus (chickenpox)	30,684	9	9	9	10	13	13	18	18	11	8	7	13	10	11	12.60	9.47–15.82; < 0.001
**Gastrointestinal diseases**	566,595	154	154	184	207	218	243	230	254	199	214	243	206	207	209	7.60	5.98–9.25; < 0.001
Cholera	22	0.02	0.01	0.01	0.01	0.004	0.01	0	0.01	0	0.003	0.02	0.01	0.002	0.01		
Campylobacteriosis	307,980	69	63	85	95	100	117	133	144	127	150	160	101	139	113	12.32	10.27–14.40; < 0.001
Cryptosporidiosis	35,589	14	17	10	17	22	19	12	11	10	7	8	15	9	13	− 1.31	− 7.79–5.62; 0.70
Typhoid	1412	0.5	0.7	0.5	0.5	0.4	0.6	0.7	0.8	0.3	0.07	0.7	0.6	0.2	0.5	5.02	0.06–10.21; 0.05
Paratyphoid	736	0.3	0.2	0.3	0.3	0.3	0.3	0.3	0.5	0.2	0.01	0.2	0.3	0.1	0.3	3.01	− 2.47–8.81; 0.29
Botulism	21	0	0.02	0.004	0.01	0	0.01	0	0.01	0.004	0.02	0.01	0.01	0.01	0.01		
Rotavirus	44,189	17	14	13	17	11	30	13	24	7	10	25	17	9	16	6.57	− 1.11–14.84; 0.10
Shiga-toxin producing *E.coli*	4627	0.5	0.8	0.5	0.6	1.4	2	2.3	2.6	2.2	2.4	3.2	1.3	2.3	1.7	30.33	22.05–39.18; < 0.001
Salmonellosis	153,147	49	55	69	71	74	67	57	58	47	42	40	63	45	57	1.13	− 3.06–5.51; 0.60
Shigellosis	15,455	2	2	4	4	6	7	10	12	6	2	5	6	4	6	27.78	23.99–31.67; < 0.001
Hepatitis A	2061	0.7	0.8	1	0.8	0.6	0.9	2	1	0.3	0.1	0.5	0.9	0.2	0.8	7.78	− 1.02–17.36; 0.09
Hepatitis E	410	0.1	0.1	0.2	0.2	0.2	0.2	0.2	0.2	0.1	0.04	0.1	0.2	0.1	0.2	2.79	− 2.54–8.40; 0.31
Listeriosis	776	0.4	0.3	0.3	0.3	0.4	0.3	0.3	0.2	0.2	0.2	0.2	0.3	0.2	0.3	− 6.37	− 9.54 – − 3.08; < 0.001
Haemolytic uraemic syndrome	170	0.1	0.1	0.1	0.1	0.1	0.1	0.1	0.1	0.1	0.03	0.1	0.1	0.04	0.1		
**Vector-borne diseases**	80,654	37	47	35	51	28	37	19	20	29	14	16	32	22	30	− 9.31	− 15.06 – − 3.16; 0.003
Barmah Forest Virus	10,134	8	18	3	3	1	2	1	1	3	2	1	5	2.5	4	− 33.75	− 45.69 – − 19.19; < 0.001
Ross River Virus	52,891	21	19	23	40	15	28	12	12	25	12	11	21	19	20	− 5.87	− 13.92–2.94; 0.19
Chikungunya	790	0.1	0.6	0.5	0.5	0.5	0.4	0.2	0.3	0.1	0.01	0.2	0.4	0.1	0.3	− 2.02	− 17.08–15.76; 0.81
Dengue	12,339	7	8	7	7	9	5	4	6	1	0.04	2	7	0.03	5	− 6.15	− 11.56 – − 0.40; 0.04
Flavivirus unspecified	226	0.03	0.1	0.1	0.1	0.4	0.1	0.03	0.04	0.1	0.01	0.02	0.1	0.03	0.1		
Japanese encephalitis	53	0.004	0.02	0.004	0.01	0	0.004	0	0.01	0.004	0.01	0.14	0.01	0.007	0.02		
Malaria	3204	2	2	1	1	1	1	2	2	1	0.2	0.8	2	0.6	1	− 0.38	− 4.10–3.50; 0.85
Murray Valley Encephalitis	6	0.004	0	0	0.01	0	0	0.004	0	0	0.004	0.004	0.002	0.002	0.002		
West Nile Virus/Kunjin Virus	11	0	0.01	0.004	0.004	0	0.02	0	0.004	0.004	0	0	0.005	0.002	0.004		
**Sexually transmissible infections**	1,358,281	442	442	453	460	512	553	573	594	501	475	524	504	488	502	5.04	4.16–5.92; < 0.001
Chancroid	1	0.004	0	0	0	0	0	0	0	0	0	0	0.0005		0.0004		
Chlamydia	1,020,824	368	362	370	363	392	412	420	423	356	340	362	389	348	379	2.60	1.92–3.28; < 0.001
Gonorrhoea	271,081	62	65	67	78	99	115	124	137	116	105	129	93	111	100	13.74	12–15.50; < 0.001
Syphilis	66,290	13	15	17	20	22	26	30	33	29	30	33	22	30	24	14.54	14–15.08; < 0.001
Congenital syphilis	83	0	0.03	0.01	0.02	0.01	0.03	0.03	0.02	0.1	0.1	0.1	0.02	0.1	0.03		
Donovanosis	2	0.004	0	0.004	0	0	0	0	0	0	0	0	0.001		0.0007		
**Bloodborne viral hepatitis**	171,842	73	76	72	70	76	67	66	60	51	48	47	70	50	64	− 2.52	− 3.98 – − 1.05; 0.001
Hepatitis B Virus	65,404	29	30	28	26	26	24	24	23	20	18	21	26	19	24	− 3.70	− 4.59 – − 2.18; < 0.001
Hepatitis C Virus	105,693	44	45	44	43	50	42	42	36	31	29	26	43	30	39	− 1.84	− 3.91–0.28; 0.09
Hepatitis D Virus	744	0.2	0.3	0.3	0.2	0.3	0.2	0.3	0.3	0.2	0.3	0.3	0.27	0.3	0.3	2.18	− 2.34–6.91; 0.35
Hepatitis, nec	1	0	0	0	0	0	0	0	0	0	0	0.003	0	0	0		
**Other diseases**	268,298	64	72	78	87	99	104	111	114	114	125	118	91	120	99	8.79	7.50–10.11; < 0.001
Invasive group A *Streptococcus*	1406	N/A	N/A	N/A	N/A	N/A	N/A	N/A	N/A	N/A	1	5			3		
Leprosy	118	0.04	0.1	0.04	0.1	0.1	0.04	0.03	0.04	0.02	0.1	0.02	0.1	0.04	0.04		
Tularaemia	2	0	0	0	0	0	0	0	0	0.01	0	0		0.005	0.0009		
Brucellosis	205	0.1	0.1	0.1	0.1	0.1	0.1	0.1	0.04	0.1	0.1	0.1	0.1	0.1	0.1		
Leptospirosis	1487	0.5	0.4	0.4	0.3	0.6	0.7	0.6	0.4	0.4	1.1	0.7	0.5	0.8	0.5	4.70	− 3.76–13.91; 0.29
Q fever	5494	2	2	2	3	2	2	2	2	2	2	2	2	2	2	1.90	− 1.25–5.15; 0.24
Congenital rubella	4	0.01	0.004	0	0.004	0	0	0	0	0	0	0	0.002		0.002		
Tetanus	44	0.03	0.02	0.01	0.01	0.03	0.02	0.01	0.01	0.03	0.01	0.004	0.02	0.02	0.02		
Varicella zoster virus (shingles)	104,293	20	22	24	27	31	38	57	60	64	44	36	35	54	38	19.65	16.32–23.07; < 0.001
Varicella zoster virus (unspecified)	154,963	41	48	52	57	64	64	51	52	48	78	75	54	63	58	2.91	− 0.95–6.92; 0.14
Monkeypox Virus	143	N/A	N/A	N/A	N/A	N/A	N/A	N/A	N/A	N/A	N/A	0.6			0.6		
Australian bat lyssavirus	1	0	0.004	0	0	0	0	0	0	0	0	0	0.0005		0.0004		
Yersiniosis	138	0.03	0.01	0.1	0.1	0.1	0.04	0.1	0.04	0.1	0.1	0.04	0.1	0.1	0.1		
**Imported diseases**	23,622	11	13	13	11	13	9	9	11	3	0.5	5	11	2	9	− 3.04	− 6.69–0.76; 0.12

Incidence: 100,000 per population per year

Average annual incidence change: percent per year

Average annual change in incidence calculated for time period from 2012 to 2019 to provide a pre-COVID-19 pandemic trend; the average annual change in incidence for ‘All diseases (includes COVID-19)’ and ‘all diseases (excludes COVID-19)’ will be the same as COVID-19 only became notifiable in 2020

Diseases with zero notifications: Yellow fever, rabies, anthrax, Middle Eastern respiratory syndrome (MERS), severe acute respiratory syndrome (SARS), poliomyelitis, viral haemorrhagic fever, lyssavirus unspecified, plague, smallpox, and avian influenza in humans

Average annual change in incidence calculated for diseases with ≥ 400 notifications over the study period

Imported diseases include cholera, typhoid, paratyphoid, hepatitis A, hepatitis E, Japanese encephalitis, malaria, dengue, chikungunya, measles, flavivirus (unspecified)

NB: overlap of individual diseases within the ‘imported’ disease group with other disease groups

*Abbreviations: N/A* Data not available as the disease is not notifiable in that year, *95% CI* 95% confidence interval, nec not otherwise specified

**Fig. 1:**
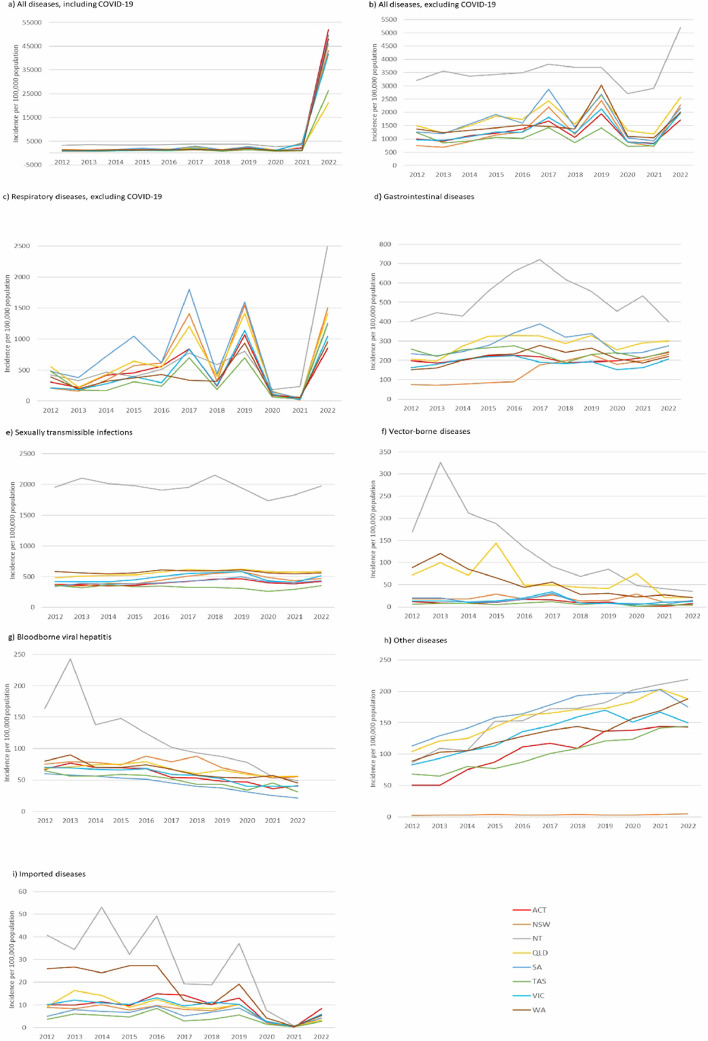
Notification incidence of disease groups by jurisdiction, Australia 2012–2022

**Fig. 2:**
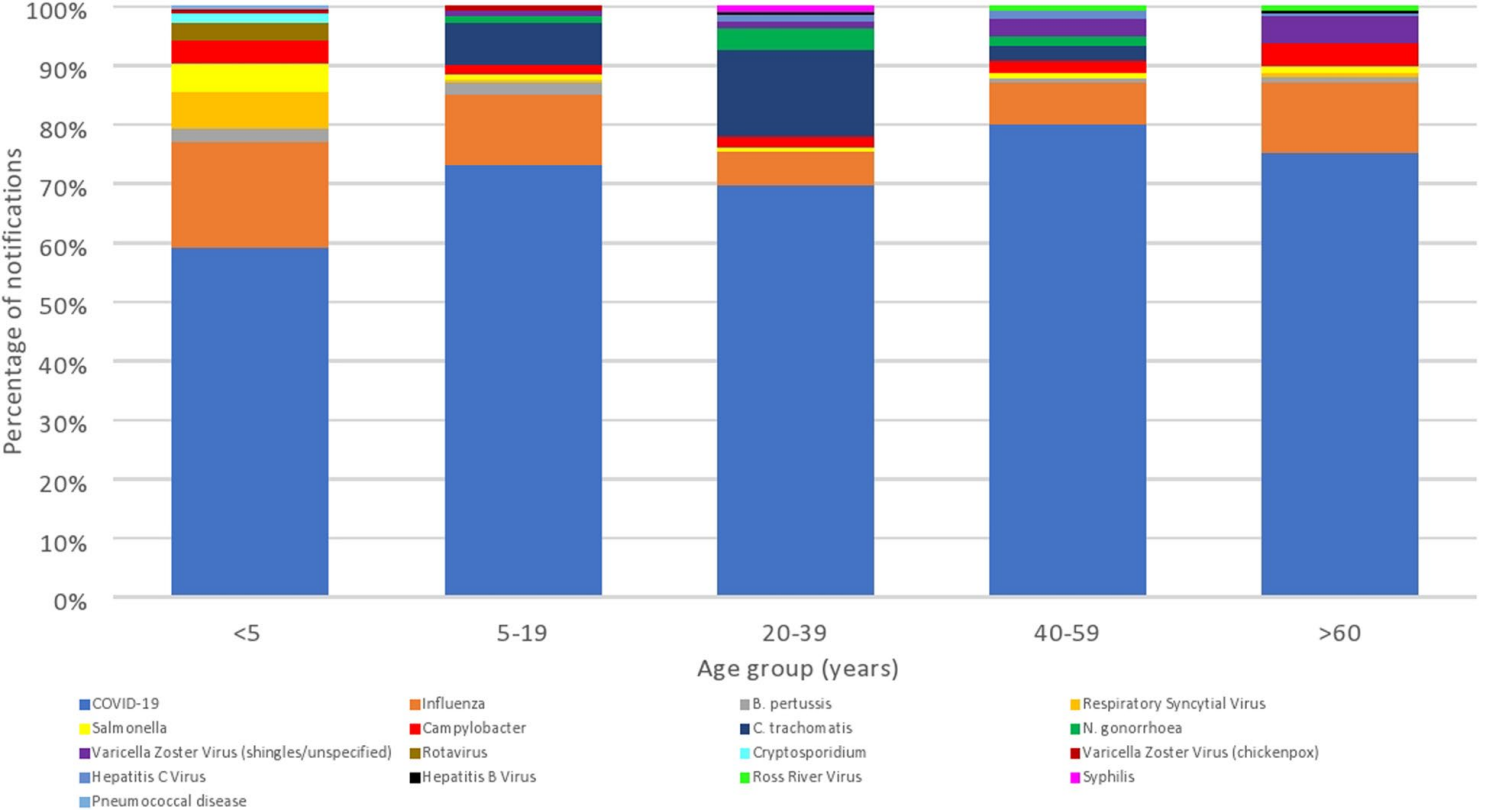
Top ten notifiable diseases by age group, Australia 2012–2022

Females comprised 50% and males 45% of notifications (5% unknown/missing). More cases of bloodborne viral hepatitis, sexually transmissible infections and gastrointestinal diseases, were notified in males at 62%, 53%, and 52%, respectively (Table 1). New South Wales (NSW) had the greatest number of notifications (4,794,516 notifications; 34%), but the Northern Territory (NT) had the highest annual notification incidence (7279/100,000/year) (Fig. 1, Table 1). Notifications were most common among the 20–39-year age group (37%) and lowest for the < 5-year age group (5%; Table 1), with the mean incidence lowest in the ≥ 60-year age group (Additional file 1: Table S3). COVID-19 and influenza were among the ten highest-incidence diseases in all age groups (Fig. 2, Additional file 1: Table S3). In young children, RSV, pertussis, and gastrointestinal diseases were also common, whereas sexually transmissible infections (chlamydia and gonorrhoea) were common in adolescents and adults, and VZV (shingles and unspecified combined) in older age groups (Fig. 2, Additional file 1: Table S3).

**Fig. 3:**
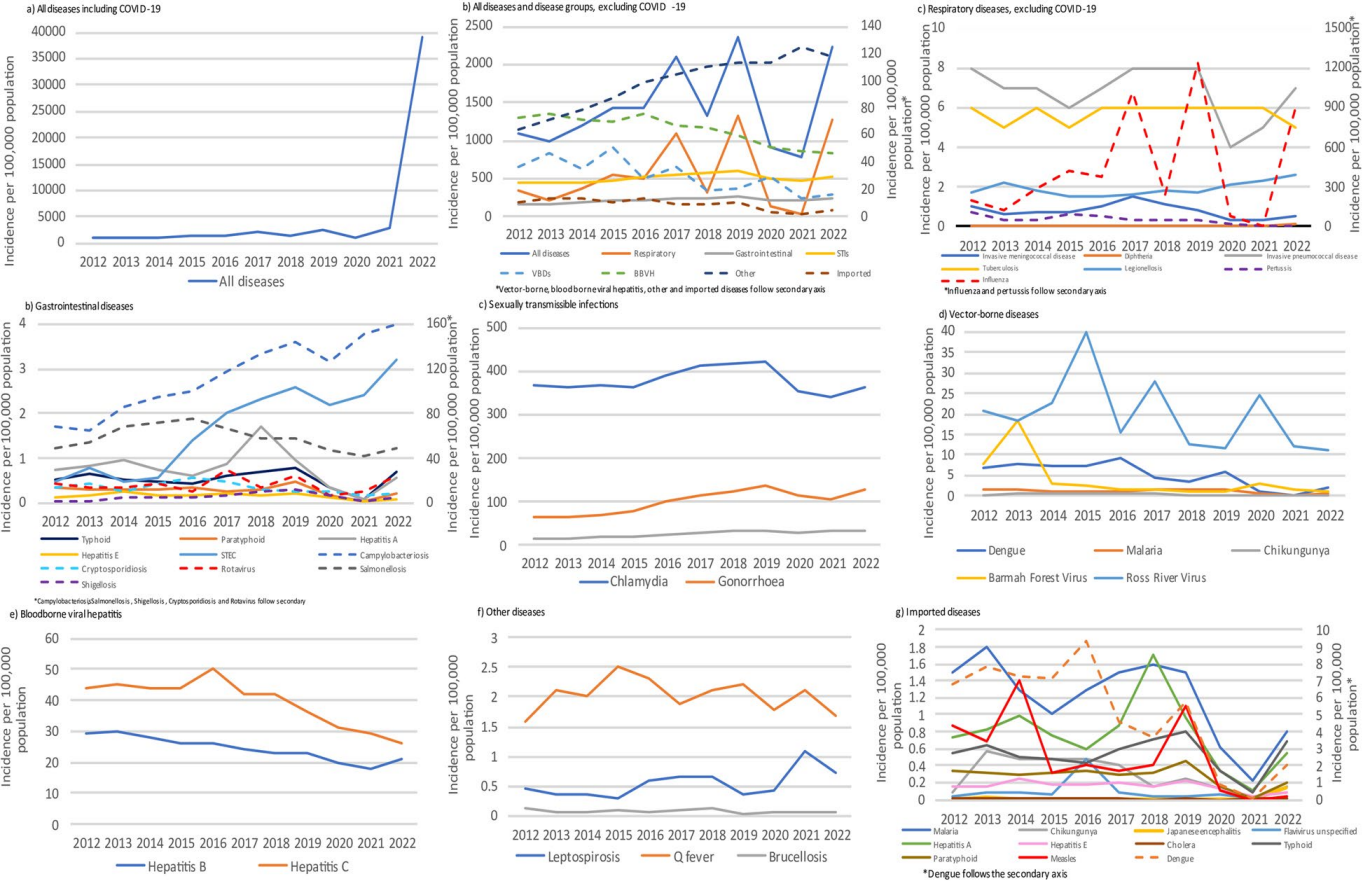
Notification incidence for all diseases, disease groups, and notable individual diseases, Australia 2012–2022

The national annual notification incidence increased by an average of 11% per year from 2012 to 2019, rising from 1100/100,000/year in 2012 (249,033 notifications) to 2363/100,000/year in 2019 (598,901 notifications). A dip in notifications in 2020 (1132/100,000/year) preceded a marked increase in notifications in 2021–2022, largely driven by COVID-19 (Fig. 3, Table 2).

### Trends from 2012 to 2019

From 2012 to 2019, the notification incidence of gastrointestinal, respiratory, sexually transmissible infections, and ‘other’ diseases increased, whereas the notification incidence of bloodborne viral hepatitis, vector-borne diseases, and imported diseases decreased (Fig. 3, Table 2). Notification incidence of respiratory diseases increased by an average of 23% per year, with influenza an important driver due to two particularly high-incidence years (2017 and 2019) and an average annual increase in incidence of 30% per year from 2012 to 2019 (Table 2). Notable increases in average annual incidence were also demonstrated for invasive pneumococcal (3% per year) and meningococcal disease (5% per year), whereas pertussis, measles, and legionellosis declined in incidence by 8%, 5%, and 2% per year, respectively. Most gastrointestinal diseases also increased in notification incidence, particularly Shiga-toxin-producing *E. coli*, shigellosis, campylobacteriosis, and hepatitis A which rose by 30%, 28%, 12%, and 8% per year, respectively (Table 2). In contrast, notification incidence of cryptosporidiosis and listeriosis declined (1% and 6% per year, respectively). Notification incidence for all vector-borne diseases decreased during this time period, with Barmah Forest virus (BFV) declining the most (33% per year). Imported vector-borne diseases, such as dengue and chikungunya also demonstrated a decrease in annual incidence (6% and 2% per year, respectively), whereas malaria remained stable (Table 2). All sexually transmissible infections increased in notification incidence at an average increase of 5% per year, with incidence peaking in 2019 (Fig. 3, Table 2). Syphilis demonstrated the largest increase of 15% per year. A 3% per year decrease in average annual notification incidence of bloodborne viral hepatitis from 2012 to 2019 was noted, with the incidence of hepatitis B and hepatitis C infections declining at an average of 4% and 2% per year, respectively. Other diseases that increased in incidence during 2012–2019 included VZV (shingles/unspecified; average increase of 23% per year) and leptospirosis (average increase of 5% per year; Table 2).

### Trends from 2020 to 2022

During the first 2 years of the COVID-19 pandemic (2020–2021), mean notification incidence of most non-COVID-19 respiratory diseases decreased compared to the 2012–2019 period; for example, influenza decreased from 487/100,000/year in 2012–2019 to 43/100,000/year in 2020–2021 (Table 2). A rebound was observed for most diseases in 2022, including influenza, invasive pneumococcal disease, and diphtheria (Fig. 3, Table 2). Exceptions to this pattern were legionellosis and ornithosis for which increased notifications were observed in 2020–2022 (Fig. 3, Table 2). Varying trends in incidence were seen for gastrointestinal infections compared to previous years: campylobacteriosis and Shiga-toxin-producing *E. coli* increased, infections that are largely imported (typhoid, paratyphoid, hepatitis A, and hepatitis E) as well as rotavirus and shigellosis decreased in 2020–2021 and then rebounded in 2022. Some other non-imported gastrointestinal diseases (e.g. cryptosporidiosis and salmonellosis) decreased with no rebound in 2022 (Fig. 3, Table 2). Notification incidence of vector-borne diseases continued to decline in 2020 and 2021 (mean incidence 22/100,000/year compared to 32/100,000/year in 2012–2019) (Fig. 3, Table 2), with imported vector-borne diseases (dengue, chikungunya, malaria) demonstrating marked decreases (Fig. 3, Table 2) followed by an increase in incidence in 2022, although still at rates below those seen prior to 2020. While sexually transmissible infection notification incidence remained fairly stable throughout this period compared to 2012–2019, bloodborne viral hepatitis notification incidence decreased from 2020 to 2022. Other notable trends included a marked increase in leptospirosis and Q-fever notification incidence in 2021 compared to previous years and ongoing increases in VZV (shingles/unspecified) notification incidence throughout 2020–2021 (Fig. 3, Table 2). The notification incidence of Japanese encephalitis (JE) also increased markedly in 2022 (0.14/100,000/year) compared to previous years (Table 2).

**Fig. 4:**
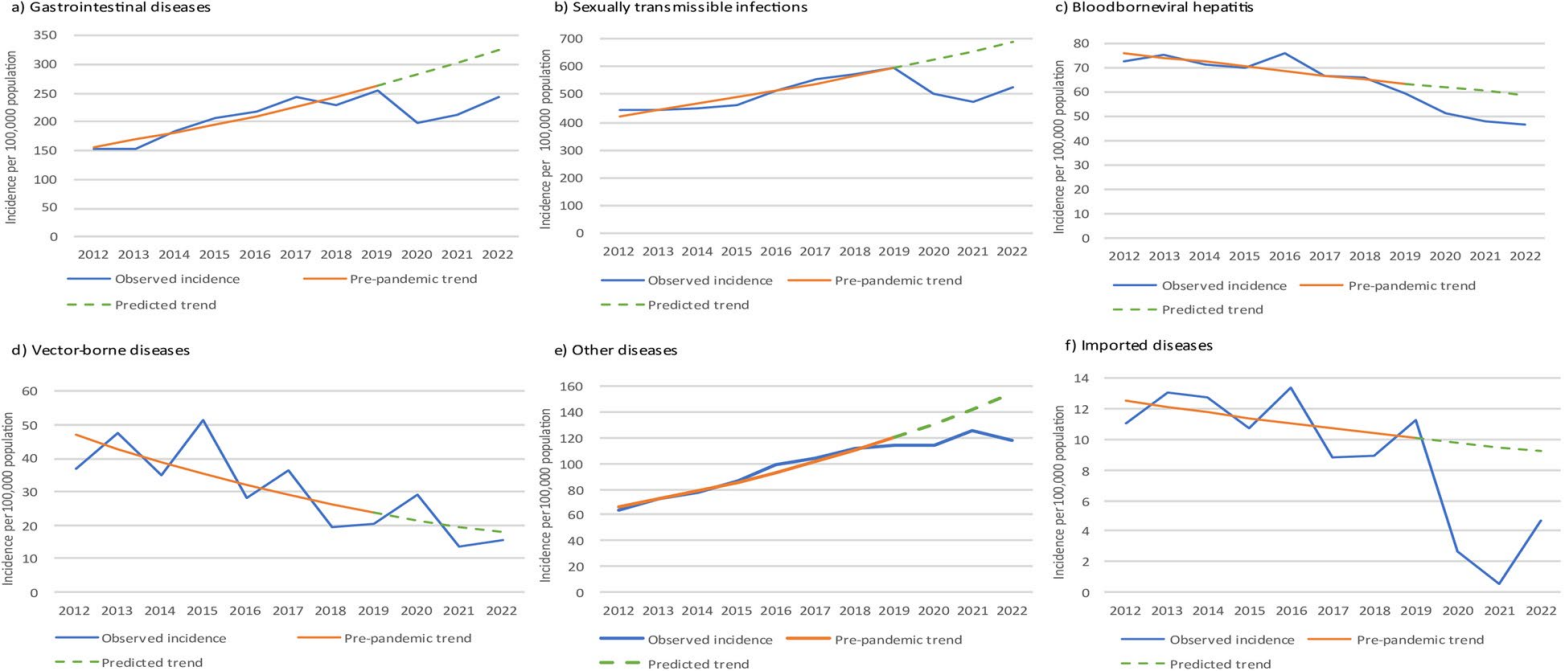
Observed notification incidence, pre-pandemic trend, and predicted trend for disease groups, Australia 2012–2022. Legend: Trends for respiratory diseases are difficult to establish due to significant seasonal variations in influenza notifications

The observed pre-pandemic trends in notification incidence for individual disease groups from 2012 to 2019 and the subsequent predicted trends from 2020 to 2022 are shown in Fig. 4. A pre-pandemic trend for respiratory diseases was difficult to establish due to significant seasonal variation in influenza cases, which dominate pre-COVID respiratory notifications. While an estimation of the number of respiratory notifications averted was not done due to difficulties in establishing a pre-pandemic baseline, notably, influenza notifications decreased from an average of 119,450 notifications per year from 2012 to 2019 to 85,136 notifications per year from 2020 to 2022. Based on pre-pandemic trends, we estimate that 65,775 gastrointestinal disease notifications, 120,821 sexually transmissible infection notifications, 9119 bloodborne viral hepatitis notifications, 5289 imported disease notifications, 87 vector-borne disease notifications, and 17,964 ‘other’ disease notifications were averted during the pandemic.

## Discussion

We provide an overview of disease trends for all nationally notifiable diseases in Australia (except HIV and CJD) from 2012 to 2022, including the impacts of the COVID-19 pandemic and associated public health measures. Case notifications increased steadily from 2012 to 2019, with a sharp rise from 2020 due to a high number of COVID-19 notifications, particularly during the delta and omicron waves in late 2021 and 2022. Excluding COVID-19 notifications, 3,962,383 notifications were received during the study period, a greater than 60% increase in notifications compared to the preceding 21-year period (1991 to 2011; 2,421,134 notifications) [1]. Multiple factors have likely contributed to this increase, including the addition of eight diseases (e.g. RSV) to the NNDL, true changes in disease incidence, new diagnostic methods, increased uptake of testing in response to awareness, and screening campaigns (e.g. for sexually transmissible infections) and growth in international travel prior to the COVID-19 pandemic.

We found an average annual increase in the incidence of all notifiable diseases of 11% per year from 2012 to 2019. The onset of the COVID-19 pandemic in 2020 and subsequent public health measures including international border closures, jurisdictional lockdowns resulting in reduced movement within and between jurisdictions, capacity limits on public venues, work and study from home, and mandatory mask-wearing had a variable impact on notification rates across different disease groups. Notification rates also varied across jurisdictions for some disease groups; for example, in 2020, non-COVID-19 respiratory diseases did not decline in incidence as much in the NT as for other jurisdictions, potentially related to an absence of community transmission of SARS-CoV-2 in the NT and a consequent lower stringency of public health restrictions [16]. Decreases in incidence were observed for imported diseases, most non-COVID-19 respiratory diseases and gastrointestinal diseases throughout 2020–2021, whereas increases in incidence were observed for diseases such as legionellosis, leptospirosis, and VZV (shingles/unspecified) where transmission is not likely to have been influenced by pandemic-related public health measures or in the case of VZV (shingles/unspecified), cases may have been triggered by COVID-19 infection.

Based on pre-pandemic trends, we estimated that COVID-19-related public health measures had a substantial impact on notifiable disease rates, resulting in a considerable number of averted notifications, in particular for sexually transmissible infections and gastrointestinal diseases. This assumption may not be valid for some diseases such as influenza which have significant seasonal variations [17]. For all disease groups, the number of notifications in 2022 had not yet reached predicted levels and it will be interesting to monitor this trend over the next few years. Our study highlights some important epidemiological trends. Respiratory diseases were the most common notifiable disease group in all jurisdictions. COVID-19 and influenza were the most commonly notified diseases overall, accounting for 80% of all notifications. Influenza notification rates were particularly high in 2017 and 2019, aligning with reports of increased influenza-related hospitalisations and deaths in these years [18] and reflecting global trends [19]. Contributing factors include low vaccine effectiveness in 2017, an earlier start and longer season in 2019 [18] and increased testing in the community [20].

Similar to other countries [7–11], the notification incidence of most non-COVID respiratory diseases decreased markedly in 2020 due to the implementation of wide-ranging non-pharmaceutical public health measures that altered behaviour and reduced human interactions. Changes in healthcare utilisation and access due to travel restrictions, healthcare facility closures, increased uptake of telehealth, and fear of contracting COVID-19 may also have led to decreased testing and diagnosis [5, 21, 22]. Following the relaxation of public health restrictions and reopening of Australia’s borders in late 2021, an increase in notification incidence for most respiratory diseases was observed in 2022, including large increases in COVID-19, influenza and RSV notifications. Ongoing community public health messaging regarding testing for influenza-like illnesses and the use of multiplex polymerase chain reaction (PCR) testing for respiratory viruses likely contributed to this increase.

The notification incidence of sexually transmissible infections increased steadily from 2012 to 2019, in keeping with a global increase of 58% in sexually transmissible infection burden since 1990 [23]. Sexually tranmissible infections were the second most commonly notified disease group overall and in the Northern Territory comprised almost 30% of notifications. Notably, we observed a marked increase in syphilis notifications in all jurisdictions (national increase of 9% per year from 2012 to 2022) which mirrors findings from other high-income settings such as the USA [24] and Western Europe [25]. Factors explaining this rise likely include an increase in high-risk sexual behaviours [24, 26] including less condom use in certain populations following the widespread roll-out of HIV pre-exposure prophylaxis (PREP) [27]. Additionally, in Australia, where a multi-jurisdictional outbreak of syphilis has led to increasing notification rates, testing has increased due to campaigns such as ‘Young, deadly, free’ [28], and ‘Don’t fool around with syphilis’ [29] and guidance for consistent routine screening of defined risk groups (e.g. antenatal testing in pregnant women) [30]. Difficulties in contact tracing in certain populations due to high mobility and certain sexual behaviours (e.g. anonymous sex, attendance at sex-on-premises venues) likely also contributed.

Notably, sexually transmissible infection notification incidence did not decrease markedly in 2020–2021, with incidence remaining similar to the average incidence observed from 2012 to 2019. A large survey (*n*= 1828) of Victorian residents in 2020 demonstrated that sexual behaviours fluctuated according to restrictions, with more engagement in physically distanced activities (e.g. virtual dates) during lockdowns, and an increase in casual sex during periods of eased restrictions [31].

The marked decrease in bloodborne viral hepatitis notification incidence over the study period likely reflects the success of public health measures such as the implementation of the universal hepatitis B vaccine in the childhood national immunisation program in 2000 [32], increased accessibility and availability of direct-acting antiviral treatments which became government-funded in 2016, and needle syringe and opioid treatment programs [33].

Gastrointestinal diseases were the third most commonly reported disease group during the study period, with increases particularly observed for Shiga-toxin-producing *E. coli*, shigellosis, and campylobacteriosis between 2012 and 2019, despite the latter only becoming notifiable in NSW in 2017. This could in part be due to a shift to culture-independent diagnostic testing for bacterial enteric pathogens following the 2013 introduction of multiplex polymerase chain reaction (PCR) diagnostic assays in Australia and their subsequent widespread use [34], as seen in other countries [35]. Increased detection of Shiga-toxin-producing *E. coli* (STEC) may not necessarily reflect increased disease, as the current case definition does not require that symptoms are present. For shigellosis, outbreaks disproportionately affecting Aboriginal and Torres Strait Islander people in northern and central Australia (2017–2019) [36] and men who have sex with men (MSM) in the eastern states (2017 onwards) [37] contributed to increased reporting rates. However, we observed a decrease in shigellosis notification incidence in 2020 and 2021 which may reflect that a proportion of these cases are imported. The observed increase in notification incidence of hepatitis A in 2018 was contributed to by two hepatitis A outbreaks: one associated with imported frozen pomegranate arils and one amongst MSM [38, 39]; subsequent decreases in incidence likely reflect the implementation of vaccination programs for high-risk populations by most jurisdictions. Although 2020–2021 saw changes in eating habits due to restrictions on travel, social gatherings, and dining out, and increased time spent at home [40], the risk of foodborne infections remained, as evidenced by the high number of campylobacter notifications in 2020 and 2021. In contrast, largely imported gastrointestinal infections (e.g. typhoid) decreased in notification incidence in 2020–2021.

Overall, we observed a decrease in vector-borne disease notification incidence throughout the study, which was most marked for BFV. However, individual disease trends varied, likely reflecting both the source of acquisition (endemic vs. imported) and changing climactic conditions. The endemic vector-borne diseases BFV and Ross River virus (RRV) increased in notification incidence in 2020, with one explanation being increased rainfall resulting in increased mosquito vectors [41]. Additionally, COVID-19-related closures of fitness and leisure facilities and restrictions on indoor gatherings may have led to more people engaging in local outdoor activities such as gardening, camping, and hiking, thereby increasing the risk of exposure [42]. A similar trend was noted with tick-borne encephalitis (TBE) in Europe during 2020, with notifications increasing by 58% in Germany, thought to be partly due to increased engagement in outdoor activities [10].

Of particular note, notifications of Japanese encephalitis increased markedly in the first 6 months of 2022, reflecting the first outbreak of JE in south-eastern Australia, with the sentinel case for the outbreak occurring in 2021 [43]. The geographical distribution of this outbreak is similar to that of previous outbreaks of other endemic flaviviruses (such as Murray Valley encephalitis virus) and is associated with La Niña weather conditions which have resulted in above average rainfall and extensive flooding, attracting waterbirds (reservoir hosts) and providing conditions for increased mosquito breeding [44].

Despite a marked increase in international travel in the years prior to the COVID-19 pandemic [45], including a record number of overseas trips by Australian travellers in 2019 [46], diseases that are primarily imported decreased by an average of 3% per year from 2012 to 2019. Unsurprisingly, we observed a decrease in imported diseases in 2020–2021 in the context of COVID-19 pandemic-related international travel disruptions. Similar to imported gastrointestinal diseases, the notification incidence of imported vector-borne diseases such as dengue, chikungunya, and malaria decreased substantially in 2020. This finding was similar to that seen in other high- and upper-middle-income countries [9–11]. Although there were no notifications of measles in 2021, cases started to increase in 2022 (*n*= 7), and increased reports are expected in the context of the current global rise in cases and re-opened international borders as demonstrated by the latest Australian surveillance data showing 19 cases notified from 1 January to 11 September 2023 [47].

Other notable epidemiological findings include an ongoing reduction in notifications of invasive meningococcal disease secondary to serogroup C following the introduction of the vaccine to Australia’s National Immunisation Program in 2003 [48]. A subsequent rise in invasive meningococcal disease notifications since 2014 coincided with increases in serogroups W and Y [49], prompting states and territories to implement the meningococcal A, C, W, and Y (MenACWY) vaccination programs [49]; decreases in invasive meningococcal disease notification incidence then followed. Another notable finding was the steady decrease in ornithosis incidence from 2012 to 2019, with an increase in 2020 related to an outbreak in the Blue Mountains Local Government Area (LGA) in NSW and a significant increase in cases in the Alpine LGA in Victoria [50, 51]. Notification incidence of leptospirosis has increased by an average of 8% per year over the study period, with contributing factors including climactic conditions favouring outbreaks [52].

Our study has some limitations. Notifications represent the frequency of disease diagnosis but not necessarily incidence as notification fractions vary between diseases, jurisdictions, population subgroups, and over time. Notifications are also influenced by jurisdictional and local diagnostic, screening, case follow-up, and notification practices. Therefore, notification incidence alone cannot determine the population burden of infectious diseases. The case definitions of notifiable diseases undergo periodic revisions which will influence notification practices. Despite uniform case definitions, notification rates may be an underestimate of the number of infections especially if diseases cause mild or no symptoms. Other limitations to the analysis include the fact that diseases became notifiable at different time points since 1991, with some diseases becoming notifiable during the study period. Other diseases that were initially grouped together were separated into individual diseases which may influence notification rates. Although there is likely to be an association, we cannot demonstrate that the reported changes in incidence during the COVID-19 pandemic are causally associated with COVID-19 versus the extent to which competing priorities and increased pressure on public health personnel and laboratories explain the findings. Other factors that could affect the incidence of notifiable diseases were not assessed in detail, such as seasonal trends, effect of vaccination, and changes in the sensitivity of surveillance.

## Conclusions

Notifiable disease notifications increased markedly during 2012–2022 compared to the previous 21 years. Prior to the COVID-19 pandemic, a steady increase in most notifiable diseases was observed, with the exception of bloodborne viral hepatitis, vector-borne diseases, and imported diseases. The pandemic had a variable impact on notification rates with substantial reductions in notifications of infectious diseases transmitted via the respiratory route such as influenza, invasive meningococcal disease and respiratory syncytial virus, and imported infections. This is likely, in part, due to non-pharmaceutical public health interventions implemented to prevent and control COVID-19. However, other diseases such as sexually transmissible infections remained stable, whereas some gastrointestinal diseases such as campylobacteriosis increased significantly. COVID-19-related public health measures likely resulted in a substantial number of averted notifications, particularly for sexually transmissible infections and gastrointestinal diseases.

## Supplementary Information

**Additional file 1: Table S1. **Diseases included in the National Notifiable Disease Surveillance System (NNDSS) grouped by main mode of acquisition. **Table S2.** Number, crude incidence and demographics of notified cases by disease group and jurisdiction (excluding COVID-19), Australia 2012-2022. **Table S3.** Notification incidence (per 100,000 population per year) by disease group, individual diseases and age groups, Australia 2012-2022.

## Data Availability

The datasets generated and/or analysed during the current study are not publicly available due to the restrictions imposed by the ethics approval from the NNDSS and CDNA. These restrictions prevent the authors from sharing datasets provided by the NNDSS unless written approval is granted by the MUHREC, NNDSS, and CDNA. However, much of the data, including annual notification numbers, age groups, sex, and jurisdiction information, is accessible via the National Notifiable Disease Surveillance System dashboard, found at https://nindss.health.gov.au/pbi-dashboard/. The corresponding authors can be contacted if access to the complete NNDSS dataset utilised in our study is required, but enabling access would necessitate obtaining additional approvals from MUHREC, NNDSS, and CDNA.

## Abbreviations

ABS: Australian Bureau of Statistics
BFV: Barmah forest virus
CDNA: Communicable Disease Network of Australia
CJD: Creutzfeldt Jacob disease
COVID-19: Coronavirus disease-2019
HIV: Human immunodeficiency virus
JE: Japanese encephalitis
LGA: Local government area
MSM: Men who have sex with men
MUHREC: Monash University Human Research and Ethics Committee
NNDL: National Notifiable Disease List
NNDSS: National Notifiable Disease Surveillance System
NSW: New South Wales
NT: Northern territory
PCR: Polymerase chain reaction
PREP: Pre-exposure prophylaxis
RRV: Ross river virus
RSV: Respiratory syncytial virus
SARS-COV-2: Severe adult respiratory syndrome coronavirus 2
TBE: Tick-borne encephalitis
VZV: Varicella zoster virus
