# Supplementary material for: The epidemiology of notifiable diseases in Australia and the impact of the COVID-19 pandemic, 2012–2022
Source: BMC Glob Public Health. 2024 Jan 2;2:1. doi: 10.1186/s44263-023-00029-y (PMC11622879; doi:10.1186/s44263-023-00029-y)
Supplement: Supplementary file 1 — Additional file 1: Table S1. Diseases included in the National Notifiable Disease Surveillance System (NNDSS) grouped by main mode of acquisition. Table S2. Number, crude incidence and demographics of notified cases by disease group and jurisdiction (excluding COVID-19), Australia 2012-2022. Table S3. Notification incidence (per 100,000 population per year) by disease group, individual diseases and age groups, Australia 2012-2022. [file 44263_2023_29_MOESM1_ESM.docx]

**Additional file 1**

**Table S1: Diseases included in the National Notifiable Disease Surveillance System (NNDSS) grouped by main mode of acquisition**

| **Mode of transmission/ acquisition** | **Disease** | **Year*** | **Variation by jurisdiction** |
| --- | --- | --- | --- |
| Gastrointestinal | Botulism  Campylobacteriosis  Cholera  Cryptosporidiosis  Hepatitis A  Hepatitis E  Haemolytic uraemic syndrome (HUS)  Listeriosis  Paratyphoid fever^$^  Poliomyelitis  Rotavirus  Salmonellosis  Shigellosis  Shiga toxin producing *E. coli* (STEC)  Typhoid fever | 1992  1991  1991  2001  1991  1999  1999  1991  2016  1991  2016  1991  1991  1999  1991 | 2017 in NSW  2018 in ACT, VIC |
| Respiratory | Avian influenza in humans  COVID-19  Diphtheria  *Haemophilus influenzae type b*  Influenza (laboratory confirmed)  Legionellosis  Measles  Meningococcal disease (invasive)  Middle Eastern Respiratory Syndrome (MERS)  Mumps  Ornithosis  Pertussis  Pneumococcal disease (invasive)  Respiratory Syncytial Virus (RSV)  Rubella  Severe Acute Respiratory Syndrome (SARS)  Smallpox  Tuberculosis  Varicella zoster (chickenpox) | 2004  2020  1991  1991  2001  1991  1991  1991  2016  1995  1991  1991  2001  2021  1993  2003  2004  1991  2006 | 2022 in NSW, ACT, TAS  Not notifiable in NSW |
| Sexually transmissible | Chancroid  Chlamydial infection  Donovanosis  Gonococcal infection  Syphilis^#^  Syphilis < 2 years  Syphilis > 2 years or unspecified duration  Congenital syphilis | 1991  1994  1991  1991  1991  2004  2004  1991 |  |
| Blood borne | Hepatitis B (newly acquired)  Hepatitis B (unspecified)  Hepatitis C (newly acquired)  Hepatitis C (unspecified)  Hepatitis D  Hepatitis (not elsewhere classified) | 1993  1991  1993  1995  1999  1991 |  |
| Vector borne | Barmah Forest Virus  Chikungunya  Dengue  Flavivirus unspecified^&^  Japanese encephalitis  Malaria  Murry Valley encephalitis  Plague  Ross River Virus  West Nile/Kunjin Virus  Yellow fever | 1995  2008  1991  2001  2001  1991  2001  1991  1993  2001  1991 |  |
| Other  Eg. Zoonoses  Direct contact  Mother to child | Anthrax  Australian bat lyssavirus  Brucellosis  Congenital rubella  Invasive group A streptococcus  Leprosy  Leptospirosis  Lyssavirus infection (not elsewhere classified)  Monkeypox Virus  Q fever  Rabies  Tularaemia  Tetanus  Varicella zoster (shingles)  Varicella zoster (unspecified)  Viral haemorrhagic fever  Yersiniosis | 2001  2001  1991  1991  2021  1991  1991  2001  2022  1991  1991  2003  1991  2006  2006  1991  1991 | Notified as lyssavirus in ACT  Notified as lyssavirus in ACT  Not notifiable in NSW  Not notifiable in NSW |
| Imported diseases^%^ | Cholera  Typhoid  Paratyphoid  Hepatitis A  Hepatitis E  Poliomyelitis  Measles  Middle-Eastern Respiratory Syndrome (MERS)  Malaria  Dengue  Chikungunya  Flavivirus unspecified  Japanese encephalitis  Yellow fever  Viral haemorrhagic fever  Rabies | **Overlap group** | |
|  |  | Gastrointestinal  Gastrointestinal  Gastrointestinal  Gastrointestinal  Gastrointestinal  Gastrointestinal  Respiratory  Respiratory  Vector borne  Vector borne  Vector borne  Vector borne  Vector borne  Vector borne Vector borne  Other | |

Abbreviations: ACT: Australian Capital Territory, NSW: New South Wales, TAS: Tasmania, VIC: Victoria

Number of notifiable diseases increased from 60 in 2011 to 68 in 2022

* Year became nationally notifiable – listed as 1991 for diseases that were nationally notifiable when NNDSS began in 1991; diseases introduced after 1991 might have cases notified to NNDSS prior to becoming nationally notifiable

^$^Paratyphoid separated from Salmonellosis in 2016

^#^Includes syphilis <2 years and >2 years/unknown duration until 2004

^&^Changed from arbovirus NEC; includes Zika virus, St. Louis encephalitis

^%^The diseases that appear in this group also appear in other disease groups, as indicated in parentheses

**Table S2: Number, crude incidence and demographics of notified cases by disease group and jurisdiction (excluding COVID-19), Australia 2012-2022**

|  | **Notifications** | | **Male** | **Age group (years)** | | | | | | | **Crude incidence (100,000 per year)**  **2012- 2022** | |
| --- | --- | --- | --- | --- | --- | --- | --- | --- | --- | --- | --- | --- |
|  | **N** | **(%)** | **(%)** | **<5 (%)** | **5-19 (%)** | | **20-39 (%)** | **40-59 (%)** | **≥60 (%)** | **Mean** | | **Range** |
| **All notifications** | 3,962,383 | 100 | 50 | 8 | 18.7 | | 40.8 | 18.1 | 14.4 | 1,462 | | 909 – 2,363 |
| **Disease group** |  | | | | |  |  |  |  |  |  |  |
| Gastrointestinal | 566,595 | 14.3 | 52 | 18.4 | 15.1 | | 26.5 | 19.2 | 20.8 | 209 | | 154 - 254 |
| Respiratory | 1,516,713 | 38.3 | 46 | 13.8 | 26.4 | | 21.9 | 18.6 | 19.3 | 557 | | 33 – 1,321 |
| Sexually transmissible infection | 1,358,281 | 34.3 | 53 | <0.5 | 16.2 | | 70.9 | 11.4 | 1.5 | 502 | | 442 - 594 |
| Bloodborne viral hepatitis | 171,842 | 4.3 | 62 | 0.2 | 2.6 | | 47.4 | 38.1 | 11.7 | 64 | | 47 - 76 |
| Vector-borne disease | 80,654 | 2 | 48 | 0.3 | 6.1 | | 32.9 | 40.5 | 20.2 | 30 | | 14 - 51 |
| Other | 268,298 | 6.8 | 46 | 1.1 | 9.1 | | 23.8 | 28 | 37.9 | 99 | | 63 - 125 |
| Imported | 23,622 | 0.6 | 55 | 3.3 | 13.4 | | 42.7 | 30.1 | 10.5 | 9 | | 0 – 13 |
| **Jurisdiction** |  | | | | |  |  |  |  |  |  |  |
| Australian Capital Territory | 57,482 | 1.5 | 51 | 7.1 | 17.1 | | 43.8 | 18.3 | 13.7 | 1,177 | | 836 – 1,946 |
| New South Wales | 1,138,171 | 28.7 | 53 | 8.6 | 20.5 | | 40.5 | 17.9 | 12.5 | 1,249 | | 685 – 2,452 |
| Northern Territory | 95,949 | 2.4 | 47 | 9.5 | 22.9 | | 46.1 | 15.5 | 6 | 3,360 | | 2,703 – 5,197 |
| Queensland | 967,087 | 24.4 | 48 | 7.7 | 19.2 | | 39.2 | 18.3 | 15.6 | 1,679 | | 1,180 – 2,645 |
| South Australia | 323,578 | 8.2 | 48 | 8.6 | 19.9 | | 34 | 18.2 | 19.3 | 1,577 | | 926 – 2,871 |
| Tasmania | 65,638 | 1.7 | 46 | 5.5 | 19.7 | | 36.9 | 17.9 | 20 | 999 | | 725 – 2,021 |
| Victoria | 902,069 | 22.8 | 52 | 5.7 | 15.8 | | 44.7 | 18.9 | 14.9 | 1,222 | | 815 – 2,144 |
| Western Australia | 412,410 | 10.3 | 49 | 5.8 | 18.5 | | 44.6 | 18 | 13.1 | 1,327 | | 1,037 – 2,032 |

Sex missing data: 0.3% for all notifications, respiratory diseases, STIs; 0.7% for Victoria

NB: Overlap of individual diseases within the ‘imported’ disease group with other disease groups

**Table S3: Notification incidence (per 100,000 population per year) by disease group, individual diseases and age groups, Australia 2012-2022**

|  | **Mean incidence by age group (2012-2019)** | | | | | **Mean incidence by age group (2012- 2022)** | | | | |
| --- | --- | --- | --- | --- | --- | --- | --- | --- | --- | --- |
|  | **< 5 years** | **5-19 years** | **20-39 years** | **40-59 years** | **≥60 years** | **< 5 years** | **5-19 years** | **20-39 years** | **40-59 years** | **≥60 years** |
| **All diseases (includes COVID-19)** | 1,680 | 1,477 | 2,103 | 1,065 | 1,069 | 4,572 | 5,167 | 5,259 | 4,739 | 3,414 |
| **All diseases (excludes COVID-19)** | 1,680 | 1,477 | 2,103 | 1,065 | 1,069 | 1,878 | 1,477 | 2,094 | 1,035 | 1,010 |
| **Respiratory diseases (includes COVID-19)** | 1,039 | 820 | 440 | 449 | 608 | 3,932 | 4,486 | 1,075 | 4,110 | 2,922 |
| **Respiratory diseases (excludes COVID-19)** | 1.039 | 820 | 440 | 449 | 608 | 1,238 | 795 | 426 | 406 | 518 |
| COVID-19 | N/A | N/A | N/A | N/A | N/A | 9,878 | 13,531 | 16,218 | 13,582 | 8,814 |
| H. influenzae type b | 0.52 | 0.06 | 0.02 | 0.04 | 0.09 | 0.51 | 0.05 | 0.02 | 0.04 | 0.08 |
| Influenza | 843 | 633 | 381 | 381 | 530 | 799 | 624 | 363 | 337 | 430 |
| Legionellosis | 0 | 0.05 | 0.43 | 2 | 5 | 0 | 0.05 | 0.44 | 2 | 6 |
| Ornithosis | 0.008 | 0.01 | 0.08 | 0.22 | 0.26 | 0.01 | 0.01 | 0.08 | 0.25 | 0.30 |
| Pertussis | 131 | 141 | 35 | 50 | 47 | 98 | 105 | 26 | 38 | 35 |
| Invasive pneumococcal disease | 15 | 3 | 3 | 7 | 16 | 15 | 3 | 3 | 6 | 15 |
| Respiratory Syncytial Virus | N/A | N/A | N/A | N/A | N/A | 1,550 | 135 | 77 | 76 | 125 |
| Diphtheria | 0 | 0.01 | 0.02 | 0.03 | 0.03 | 0.02 | 0.03 | 0.03 | 0.03 | 0.03 |
| Tuberculosis | 1 | 2 | 10 | 5 | 6 | 1 | 2 | 10 | 5 | 6 |
| Invasive meningococcal disease | 4 | 1 | 0.69 | 0.46 | 1 | 3 | 1 | 0.60 | 0.40 | 0.72 |
| Measles | 2 | 1 | 1 | 0.26 | 0.01 | 1 | 0.72 | 0.82 | 0.20 | 0.01 |
| Mumps | 1 | 3 | 3 | 1 | 0.52 | 1 | 3 | 2 | 1 | 0.43 |
| Rubella | 0.05 | 0.04 | 0.17 | 0.06 | 0.02 | 0.04 | 0.03 | 0.13 | 0.05 | 0.02 |
| Varicella zoster virus (chickenpox) | 41 | 36 | 7 | 3 | 2 | 36 | 33 | 6 | 3 | 2 |
| **Gastrointestinal diseases** | 619 | 192 | 192 | 153 | 196 | 615 | 171 | 195 | 157 | 209 |
| Cholera | 0.02 | 0 | 0.01 | 0.01 | 0.01 | 0.02 | 0 | 0.01 | 0.01 | 0.01 |
| Campylobacteriosis | 160 | 77 | 100 | 88 | 121 | 179 | 88 | 112 | 98 | 138 |
| Cryptosporidiosis | 78 | 17 | 17 | 6 | 3 | 66 | 15 | 15 | 5 | 3 |
| Typhoid | 1 | 0.79 | 1 | 0.24 | 0.07 | 1 | 1 | 1 | 0.20 | 0.07 |
| Paratyphoid | 0.31 | 0.33 | 0.66 | 0.19 | 0.07 | 0.25 | 0.28 | 0.54 | 0.2 | 0.06 |
| Botulism | 0.10 | 0 | 0 | 0.002 | 0 | 0.11 | 0 | 0.001 | 0.001 | 0 |
| Rotavirus | 134 | 13 | 7 | 6 | 14 | 132 | 12 | 7 | 5 | 12 |
| Shiga-toxin producing E.coli | 3 | 1 | 1 | 1 | 2 | 3 | 1 | 1 | 1 | 3 |
| Salmonellosis | 225 | 52 | 56 | 44 | 51 | 217 | 48 | 50 | 40 | 48 |
| Shigellosis | 15 | 4 | 8 | 6 | 3 | 15 | 4 | 7 | 5 | 3 |
| Hepatitis A | 1 | 1 | 1 | 0.73 | 0.34 | 0.80 | 1 | 1 | 0.58 | 0.27 |
| Hepatitis E | 0.01 | 0.04 | 0.28 | 0.20 | 0.20 | 0.01 | 0.03 | 0.24 | 0.16 | 0.17 |
| Listeriosis | 0.37 | 0.006 | 0.13 | 0.15 | 1 | 0.33 | 0.01 | 0.12 | 0.14 | 1 |
| Haemolytic uraemic syndrome | 0.26 | 0.08 | 0.03 | 0.02 | 0.06 | 0.37 | 0.07 | 0.03 | 0.02 | 0.07 |
| **Vector-borne diseases** | 2 | 13 | 41 | 53 | 32 | 2 | 10 | 35 | 47 | 30 |
| Barmah Forest Virus | 0.10 | 2 | 5 | 7 | 5 | 0.10 | 1 | 4 | 6 | 4 |
| Ross River Virus | 0.59 | 6 | 23 | 34 | 23 | 0.45 | 5 | 21 | 32 | 22 |
| Chikungunya | 0.03 | 0.11 | 0.49 | 0.61 | 0.25 | 0.04 | 0.10 | 0.39 | 0.49 | 0.21 |
| Dengue | 0.68 | 3 | 10 | 9 | 4 | 0.52 | 2 | 7 | 7 | 3 |
| Flavivirus unspecified | 0.008 | 0.03 | 0.17 | 0.16 | 0.05 | 0.01 | 0.02 | 0.13 | 0.13 | 0.05 |
| Japanese encephalitis | 0 | 0.003 | 0.004 | 0.01 | 0.01 | 0.01 | 0.01 | 0.02 | 0.04 | 0.04 |
| Malaria | 0.59 | 1 | 2 | 2 | 0.58 | 0.50 | 1 | 2 | 1 | 0.48 |
| Murray Valley Encephalitis | 0.008 | 0.006 | 0.002 | 0 | 0.002 | 0.006 | 0.004 | 0.001 | 0 | 0.003 |
| West Nile Virus/Kunjin Virus | 0 | 0 | 0.007 | 0.006 | 0.007 | 0 | 0 | 0.01 | 0.004 | 0.005 |
| **Sexually transmissible infections** | 3 | 473 | 1,236 | 210 | 36 | 3 | 442 | 1,248 | 222 | 37 |
| Chancroid | 0 | 0.003 | 0 | 0 | 0 | 0 | 0.002 | 0 | 0 | 0 |
| Chlamydia | 2 | 414 | 971 | 119 | 15 | 2 | 383 | 958 | 123 | 15 |
| Gonorrhoea | 1 | 56 | 225 | 64 | 8 | 1 | 54 | 244 | 70 | 8 |
| Syphilis | 0.02 | 4 | 40 | 27 | 13 | 0.04 | 5 | 46 | 32 | 13 |
| Congenital syphilis | 0.43 | 0 | 0 | 0 | 0 | 0.49 | 0 | 0 | 0 | 0 |
| Donovanosis | 0 | 0 | 0.004 | 0 | 0 | 0 | 0 | 0.003 | 0 | 0 |
| **Bloodborne viral hepatitis** | 2 | 10 | 119 | 103 | 35 | 2 | 9 | 107 | 95 | 36 |
| Hepatitis B Virus | 1 | 5 | 48 | 33 | 15 | 0.64 | 4 | 43 | 32 | 16 |
| Hepatitis C Virus | 1 | 5 | 71 | 70 | 20 | 1 | 5 | 64 | 63 | 20 |
| Hepatitis D Virus | 0 | 0.03 | 0.43 | 0.44 | 0.14 | 0 | 0.03 | 0.41 | 0.46 | 0.17 |
| **Other diseases** | 16 | 52 | 76 | 97 | 163 | 18 | 49 | 82 | 108 | 181 |
| Invasive group A Streptococcus | N/A | N/A | N/A | N/A | N/A | 6 | 2 | 2 | 3 | 4 |
| Leprosy | 0 | 0.009 | 0.11 | 0.05 | 0.02 | 0 | 0.01 | 0.10 | 0.04 | 0.02 |
| Tularaemia | 0 | 0 | 0 | 0.004 | 0 | 0 | 0 | 0 | 0.003 | 0 |
| Brucellosis | 0.02 | 0.03 | 0.14 | 0.10 | 0.05 | 0.02 | 0.03 | 0.13 | 0.10 | 0.05 |
| Leptospirosis | 0 | 0.20 | 0.73 | 0.62 | 0.29 | 0 | 0.22 | 0.82 | 0.73 | 0.41 |
| Q fever | 0.10 | 0.73 | 2 | 4 | 3 | 0.13 | 0.63 | 2 | 3 | 3 |
| Congenital rubella | 0.03 | 0 | 0 | 0 | 0 | 0.02 | 0 | 0 | 0 | 0 |
| Tetanus | 0.01 | 0.01 | 0.01 | 0.01 | 0.05 | 0.006 | 0.01 | 0.01 | 0.004 | 0.05 |
| Varicella zoster virus (shingles) | 4 | 13 | 26 | 36 | 73 | 3 | 12 | 29 | 42 | 81 |
| Varicella zoster virus (unspecified) | 12 | 38 | 47 | 57 | 87 | 13 | 36 | 50 | 61 | 96 |
| Monkeypox Virus | N/A | N/A | N/A | N/A | N/A | 0 | 0 | 1 | 0.65 | 0.02 |
| Australian bat lyssavirus | 0 | 0.003 | 0 | 0 | 0 | 0 | 0.002 | 0 | 0 | 0 |
| Yersiniosis | 0.10 | 0.06 | 0.07 | 0.03 | 0.02 | 0.10 | 0.06 | 0.07 | 0.04 | 0.03 |
| **Imported diseases** | 6 | 8 | 17 | 13 | 6 | 5 | 6 | 13 | 10 | 5 |

Abbreviations: N/A: Data not available as disease not notifiable between 2012 and 2019

NB: Overlap of individual diseases within the ‘imported’ disease group with other disease groups
